# Participation of traditional birth attendants in prevention of mother-to-child transmission of HIV services in two rural districts in Zimbabwe: a feasibility study

**DOI:** 10.1186/1471-2458-8-401

**Published:** 2008-12-05

**Authors:** Freddy Perez, Khin Devi Aung, Theresa Ndoro, Barbara Engelsmann, François Dabis

**Affiliations:** 1 Institut de Santé Publique, d'Epidémiologie et de Développement (ISPED), Université Victor Segalen Bordeaux 2, France; 2 Organization for Public Health Interventions and Development (OPHID), Harare, Zimbabwe

## Abstract

**Background:**

Prevention of Mother-to-Child Transmission of HIV (PMTCT) is among the key HIV prevention strategies in Zimbabwe. A decrease in use of antenatal care (ANC) services with an increase in home deliveries is affecting the coverage of PMTCT interventions in a context of accelerated economic crisis. The main objective was to evaluate acceptability and feasibility of reinforcing the role of traditional birth attendants (TBAs) in family and child health services through their participation in PMTCT programmes in Zimbabwe.

**Methods:**

A community based cross-sectional survey was undertaken using multistage cluster sampling in two rural districts through interviews and focus group discussions among women who delivered at home with a TBA, those who had an institutional delivery and TBAs.

**Results:**

45% of TBAs interviewed knew the principles of PMTCT and 8% delivered a woman with known HIV-positive status in previous year. Of the complete package of PMTCT services, more than 75% of TBAs agreed to participate in most activities with the exception of performing a blood test (17%), accompanying new-borns to closest health centre to receive medication (15%) and assisting health centres in documentation of the link ANC-PMTCT services (18%). Women who delivered at home were less likely to have received more than one ANC service or have had contact with a health centre compared to women who delivered in a health centre (91.0% vs 72.6%; P < 0.001). Also, 63.6% of the women who delivered in a health centre had the opportunity to choose the place of delivery compared to 39.4% of women who delivered at home (P < 0.001). More than 85% of women agreed that TBAs could participate in all activities related to a PMTCT programme with the exception of performing a blood test for HIV. Concerns were highlighted regarding confidentiality of the HIV-serostatus of women.

**Conclusion:**

Although the long-term goal of ANC service delivery in Zimbabwe remains the provision of skilled delivery attendance, PMTCT programmes will benefit from complementary approaches to prevent missed opportunities. TBAs are willing to expand their scope of work regarding activities related to PMTCT. There is a need to reinforce their knowledge on MTCT prevention measures and better integrate them into the health system.

## Background

In the year 2000, the member countries of the United Nations agreed to reduce the mortality rate of children younger than five years of age by two-thirds and maternal mortality rate by three quarters by 2015 as part of the Millennium Development Goals (MDGs) [1]. However, these goals are unlikely to be achieved due to the inability of current maternal and child health programmes to reach the poorest households. Achieving the health MDGs will be a major challenge in the years ahead, and yet these goals must be attained if we are to succeed in reversing the global HIV/AIDS epidemic [2].

Women account for half of the 33.2 million people living with HIV-1 in 2007. Sub-Sahara Africa remains the most affected region where more than two-thirds (68%) of HIV-infected people live with females constituting 61% of those infected [3]. Thus; high levels of maternal mortality co-exist with high levels of HIV prevalence among women of childbearing age [4]. In this context, there are important disparities with respect to access to health care for women [5]. Data from the World Bank shows that in the poorest 20% of households in most developing countries, more than 90% of deliveries take place at home [6]. Consequently, each year more than 60 million women worldwide deliver without the assistance of skilled care [7].

With such high maternal mortality rates in resource-poor settings, priority interventions through the 'Safe Motherhood Initiative' have been proposed and implemented. This public health strategy emphasises safe delivery through the provision of skilled birth attendants, improved basic obstetric services in health facilities, development of prenatal care, access to emergency obstetric care in hospitals, and family planning as key interventions to reduce neonatal and maternal mortality [8]. It is now 20 years since the Safe Motherhood initiative was launched and little progress has been reported [9,10]. Accomplishments have been achieved concerning greater knowledge about what works and what does not, but overall the reduction in maternal mortality has been modest with a decline of 5.4% between 1990 and 2005. The regions of Sub-Saharan Africa, western Asia and south Asia have shown little progress [11,12]. The stated reasons for this have included: absence of a clear focus, strategic errors such as focusing only on mother's risk of complications through screening at antenatal consultations, and an over reliance on traditional birth attendants (TBAs) [13,14].

Skilled attendance at birth is a key indicator for measuring progress towards improved women's health. Available data from developing countries show an important increase in skilled attendance at birth: from 45% to 54% between 1990 and 2000, except for the sub-Saharan Africa region, where coverage has stagnated at approximately 40% [15]. Additionally, a recent report confirms that world-wide, for the year 2007, an estimated 63% of all births were attended by a skilled health-care worker with considerable variations between developed regions (99%) as compared to developing countries (59%) [16].

In Zimbabwe, maternal mortality rates have risen to over 880 deaths per 100,000 live births [17] and HIV/AIDS is seen as one of the major contributing factors [18]. Current national data shows that although more than 90% of pregnant women reportedly attend formal health facilities for antenatal care at least once during pregnancy, more than 31% of deliveries take place outside the formal health system (an increase compared to the 1999 data recorded in 1999 of 23%) with selected provinces reporting up to 42% [19]. These deliveries are assisted by either trained, untrained TBAs or family members.

Zimbabwe has one of the greatest HIV burdens in the world with an average antenatal HIV prevalence rate of 15.6% [20]. Prevention of mother-to-child transmission of HIV (PMTCT) is among the key HIV prevention strategies in the country's national HIV/AIDS response. In spite of the important efforts and the rapid expansion of the national PMTCT programme, uptake of PMTCT remains suboptimal. Certain steps of the intervention cascade need to be substantially improved to increase the coverage of services particularly in the later stages of the intervention.

In 2006, 19 578 pregnant women were identified nation-wide as HIV-infected, of whom 60% received some form of antiretroviral (ARV) prophylaxis to prevent transmission of the virus to their babies. This translated to approximately 30% coverage of the total number of HIV positive pregnant women in need [21]. Limited ANC services with an increase in home deliveries is not only reducing access to a skilled practitioner through maternal services but also restricts the opportunities to provide PMTCT services in a context of an accelerated economic crisis [22,23], specially in rural areas [24]

Recent data shows that, in low-and middle-income countries, the proportion of HIV-positive pregnant women receiving antiretroviral prophylaxis for PMTCT in 2006 was 23% [25]. Globally, PMTCT coverage is far below what is required to meet the United Nations target of reducing the proportion of children infected with HIV by 50% in 2010 [26]. The attainment of these figures will need that 80% of all pregnant women accessing antenatal care receive services for PMTCT of HIV [27,28]. This will require strengthening of maternal and child health services as well as the health systems and the development of new interventions to improve the uptake of PMTCT services.

Antenatal care as well as deliveries in an institutional setting with skilled health workers for all women remains a distant reality. In resource-poor countries, between 60% and 90% of deliveries in rural areas are assisted by TBAs [29,30]. Preference for home births is associated with cultural norms and religious beliefs. TBAs speak the local language, have the trust of community members and can provide psychosocial support at birth [31].

Public health programmes are seeking to enhance the role of TBAs by encouraging their participation in PMTCT programmes [29,32,33]. Given the potential coverage of the underserved population, participation of TBAs has been piloted to help improve the coverage and quality of services offered to rural populations; their participation being defined by a package of activities that they are allowed to perform.

The main objective of this study was to evaluate the acceptability and feasibility of reinforcing the role of TBAs in the family and child health services through their participation in the PMTCT programmes in two rural districts of Zimbabwe.

## Methods

### Setting and sampling

A community-based cross-sectional quantitative and qualitative survey was conducted in Murewa and Goromonzi districts, two rural districts of Mashonaland East province, Zimbabwe where PMTCT services are being provided. The targeted study populations included TBAs above 18 years of age practicing in these two districts. TBAs refer to traditional, independent (of the health system) community-based health providers which include trained and untrained TBAs [34]. Trained TBAs are defined as those who have received a short-course of training through the modern health care sector to upgrade their skills [35] and were in possession of a badge or certificate which has been issued on completion of her training. The criterion for recruitment of an untrained TBA was that she should have delivered a woman not more than a year ago before the date of the survey and was not formally trained. Pregnant women in their second or third trimester of pregnancy (more than 12 weeks into pregnancy) and who also had another child; women who had an institutional delivery within the past 12 months and women who had a home birth with a TBA within the past 12 months were also included in the survey. Recruitment was based on the above-defined inclusion criteria only and was done through consecutive enrolment until the required sample was obtained.

The sample frame for this study was based on the assumptions that among approximately 2500 pregnant women in their second or third trimester of pregnancy and 2000 women who delivered at home in the two districts, during the last year, 75% would have a favourable opinion of the participation of TBAs in the PMTCT programme. For women who had delivered at a health centre, of approximately 4000 women from the two districts, 75% would have a favourable opinion of the participation of TBAs in the PMTCT programme. Finally, the assumption that 66% of trained TBAs and 33% of untrained TBAs would be willing to participate in the PMTCT programme was adopted. Calculations were made to allow a precision of 5%, with an alpha type one error of 5%; which resulted in a minimum sample size for the two districts of 188 pregnant women, 184 women who had delivered at home within the past 12 months, 193 women who had delivered at a health centre within the past 12 months, 33 trained and 33 untrained TBAs with an attempt to provide district-specific estimates for programmatic purposes. We increased the sample size by 10% to cater for anticipated refusal or non-response.

Sampling was done by a multistage stratified, random cluster sampling procedure of households in the two rural districts [36]. From the sample frame, 30 clusters of 7 women for each group of women (pregnant, those who delivered at home and those who delivered at health centre) were required from all 55 wards (administrative unit) of both districts. Within each of the districts, the clusters were selected through probability proportionate size sampling using data from the 2003 Census Office of the Central Statistics Office [37]. Using this sampling method, 15 out of 30 wards corresponding to 15 clusters were situated in Murewa district, and 14 out of 25 wards corresponding to 15 clusters were situated in Goromonzi district (one ward was linked to two clusters in this last district). In a second stage, the village for each ward where the cluster was identified was selected randomly based on data obtained from the district health information system and if necessary completed by the practicing health counsellors of each ward.

The survey was conducted from May to July 2006. Villages comprising each cluster were attended by a single survey team. Using a random process, the team leader (a medical doctor or community nurse) identified the first household to be targeted based on a list of heads of households and maps obtained from the head of the village. Thereafter, consecutive households were approached according to the pre-established protocol and eligible women in each of the categories were identified. Women were subsequently introduced to the research team, given oral explanation of the study and if agreed, recruited until the total number of participants required was enrolled.

Two questionnaires were designed to collect qualitative and quantitative data: one for women (pregnant or who had delivered during the last year) and one for TBAs, both of which were pre-tested in a nearby district (Buhera district) to translate the tool from English to Shona and refine and validate the questionnaire. For the questionnaire on women, information was systematically collected on socio-demographic variables, health seeking behaviours for the last pregnancy, knowledge on HIV/AIDS and perceptions concerning the participation of TBAs in PMTCT services. The questionnaire for TBAs included data on socio-demographic variables, their background and the trainings they had received, their scope of activities as a TBA, their knowledge, attitude and practice with regards to HIV/AIDS, and their willingness to participate in the PMTCT programme.

In addition to the community-based survey, qualitative information was generated through four focus group discussions (FGDs). For this, purposive sampling was undertaken till the necessary number was attained. Here, TBAs who had participated in the community-based survey were invited to attend the FGDs at a later stage or identified with the help of village health workers of the community. Each TBA was accompanied by a woman who they had helped to deliver if agreed and/or identified also with the help of the village health worker. Women who had delivered at a health centre were recruited among the mothers who were attending the family health clinic for immunization or growth monitoring of her child on the day of the planned focus group.

A discussion guide was designed for each of the following groups: trained TBAs (n = 8), untrained TBAs (n = 11), women who had delivered at a health institution (n = 8) and women who had delivered at home with a TBA (n = 8). Questions for TBAs in the FGDs covered information on the role that TBAs have with pregnant women in their communities regarding the preparation for the women's delivery, the assistance they provide during delivery and during the postnatal period, problems they encounter in providing these services, reasons why they think women prefer to deliver with TBAs and lastly, basic concepts on HIV/AIDS. Special attention was given to their willingness to participate in PMTCT interventions. For women, the topics covered were: what women do to prepare for their delivery, the type of health information they have access to, the services provided at the health centre and/or by TBAs, their opinion on the services that are provided by the health centre/TBAs, and their opinion on having TBAs participate in PMTCT activities. On average, the discussions lasted 90 minutes per session. A moderator (expert in social sciences) conducted the interviews in local vernacular language and a note-taker was also present.

### Data Collection and Analysis

The questionnaires were administered to the women and TBAs in the community using interviewers who were students in their final year of medicine. Six interviewers were recruited and a one-day training session was conducted on interview and sampling techniques, selection criteria of the women and TBAs and content and application of the questionnaires. During this training session, the questionnaires were translated into vernacular language.

The completed questionnaires were verified by the supervisor every other day and data was entered into a specifically designed database throughout the survey. Prior to analysis, missing data was checked against the survey forms. Quantitative and categorical data was entered and analysed in EPI-INFO 2004 (US Centres for Disease Control and Prevention, Atlanta, GA). TBA and women characteristics were described using median values and interquartile ranges (IQR) for continuous variables, and counts and percentages for categorical data. Differences of proportions were tested using a Χ^2 ^test. Qualitative data was coded and described as such.

Focus groups data was tape recorded, and notes also were taken during and immediately after each FGD. Data was transcribed and translated from Shona to English. Analysis was done by coding the information in categories based on topical questions and then compared to search for associations, patterns and identification of themes.

### Ethical Considerations

Authorisation to carry out the survey was obtained from the Ministry of Health and Child Welfare of Zimbabwe and the provincial and district administrators. Verbal informed consent was obtained and confidentiality was assured for all the participants.

## Results

### Community-based survey

Of a total of 650 women interviewed, 23 questionnaires were incomplete and were excluded from the analysis. Additionally, of a total of 78 TBAs interviewed, six were not within the age criteria and were thus excluded from the analysis. Thus a total of 627 questionnaires applied to women and 72 questionnaires applied to TBAs. Table 1 shows the distribution of women (pregnant, having delivered in a health facility or at home with a TBA) and TBAs (trained and untrained) interviewed by district.

**Table 1:** Distribution of women and traditional birth attendants interviewed by district, Murewa and Goromonzi districts, Zimbabwe, 2006

**District**	**Women who delivered at home**	**Women who delivered at a health centre**	**Pregnant women**	**Trained TBA†**	**Untrained TBA**
**Murewa**	107 (51.4%)	108 (51.7%)	103 (49.0%)	14 (46.7%)	24 (57.1%)
**Goromonzi**	101 (48.6%)	101 (48.3%)	107 (51.0%)	16 (53.3%)	18 (42.9%)
**Total**	208 (100%)	209 (100%)	210 (100%)	30 (100%)	42 (100%)

† TBA: traditional birth attendant

Table 2 shows the main socio-demographic characteristics of the group of women interviewed who delivered at home and those who delivered at a health facility. Most of the women interviewed were between the ages of 20 to 29 years (63%), the majority (88%) being currently married. Women who delivered at home had a lower level of education compared to women who delivered in a health centre (67.7% vs. 77.5%; P < 0.02 respectively). No significant correlation was found between age, marital status, religion, principal occupation of women and whether women delivered at home or at a health facility.

**Table 2:** Socio-demographic characteristics of women who delivered at home (n = 208) and at a health facility (n = 209), TBA survey, Murewa and Goromonzi districts, Zimbabwe, 2006

**Selected characteristics**	**Women who delivered at home** **(n = 208)**	**Women who delivered at a health facility** **(n = 209)**	**P-value**
**Median age (IQR)**	24 (20–29)	23 (20–26)	0.09
			
**Marital status**			
Married	86.0%	89.5%	0.30
**Level of Education**			
Secondary education or more	67.7%	77.5%	0.02
**Main Religion**			
Apostolic	29.8%	36.6%	0.09
**Principle occupation**			
Subsistence farming	50.0%	51.0%	0.62

IQR: interquartle range

All TBAs were female, their age range was 25 – 70 years; the medium age was 62 years for trained TBAs and 52 for untrained TBAs. Among the total number of TBAs interviewed trained TBAs (41%) were older (P < 0.001), and almost all were either married (53%) or widowed (39%). Untrained TBAs had become a TBA due to an emergency delivery more frequently than trained TBAs (P = 0.002) and had less experience than trained TBAs (P = 0.007). Years working as a TBA varied from one year to more than 35 years. No difference was found among the two groups regarding level of education, religion or principal occupation (Table 3). None of the TBAs had a formal job and they had overall limited education: 47% had completed primary school and 23% had no formal education.

**Table 3:** Socio-demographic characteristics of trained and untrained TBAs, TBA survey, Murewa and Goromonzi districts, Zimbabwe, 2006

**Selected characteristics**	**Trained TBAs (n = 30)**	**Untrained TBAs (n = 42)**	**P-value**
**Median age (IQR)**	62 (55–68)	52 (45–60)	< 000.1
			
**Marital status**			
Married	40.0%	61.9%	0.06
**Level of Education**			
Formal schooling	30.0%	19.0%	0.2
**Main Religion**			
Catholic	26.6%	38.0%	0.31
**Principle occupation**			
Subsistence farming	53.3%	61.9%	0.46
**How became a TBA**			
Emergency delivery	33.3%	70.7%	0.002
**Period as TBA**			
≥ 10 years	76.6.%	45.2%	0.007

TBA: traditional birth attendant; IQR: interquartle range

Women who delivered in a health centre were more likely to have received more than one ANC service or have had contacts with a health centre (91.0%) than those who delivered at home (72.6%) (P < 0.001). Of all the women who had had an antenatal contact with a health facility, the average gestational age for the first ANC visit was at six months of pregnancy in both groups. Additionally, 63.6% of the women who delivered in a health centre had had the opportunity to choose the place of delivery compared to 39.4% of women who delivered at home (P < 0.001). In this last group, place of delivery at home was decided by another member of the family (among them, husband, partner, mother and mother-in-law) for 60% of women. (Table 4). Basic knowledge on PMTCT issues was relatively high in both groups. Among a total of 292 (70%) women from both groups who responded affirmatively that HIV can be transmitted from mother to child, almost 90% thought that transmission of HIV from mother-to-child could be prevented and were able to cite a minimal of three potential interventions for PMTCT. The least known intervention by both groups was "giving medication to the new-born" (40%). No significant difference was found between the two groups. Furthermore, 61% of women who delivered at a health facility and 62% who delivered at home reported they would disclose their HIV status to a TBA.

**Table 4:** Selected obstetric and PMTCT factors related to place of delivery among women who delivered in the last year, TBA survey, Murewa and Goromonzi districts, Zimbabwe, 2006, univariate analysis.

**Selected characteristics**	**Women who delivered at home** **(n = 208)**	**Women who delivered at a health facility** **(n = 209)**	**P-value**
**Had received ANC services (> 1)/contact health centre**	72.6%	91.0%	< 0.001
**Pregnant women chose place of delivery**	39.4%	63.6%	< 0.001
**Basic knowledge on PMTCT**	68.9%	71.2%	0.52
**Knows where closest PMTCT site is located**	57.4%	64.7%	0.11
**Agree to disclosure HIV status to a TBA**	61.5%	61.2%	0.96

ANC: antenatal care; PMTCT: prevention of mother-to-child transmission of HIV; TBA: traditional birth attendant

The main health activities TBAs (trained and untrained) reported to undertake were assistance during delivery, postpartum care (follow-up of women within a week after delivery) (87%) and routinely advising women to go to a health centre after delivery (76%). Of the total of 72 TBAs interviewed only 27 (38%) had given information to women on HIV/AIDS related issues. Information and advice to women on topics such as the benefits of having an HIV test, condom use or breastfeeding counselling were seldom practiced. Few TBAs had delivered a woman with a known HIV-positive status (8%). These results showed no statistical difference between trained and untrained TBAs (data not shown).

Regarding knowledge of HIV/AIDS basic issues among TBAs in the two districts, when asked if they knew that HIV can be transmitted from mother-to-child, 69% (n = 50) gave an affirmative answer. Of this last group, only 57% (n = 21) reported knowing interventions to prevent mother-to-chid transmission of HIV, the administration of single-dose nevirapine (sdNVP) being the most known (12%). Finally, very few TBAs (less then 7% of the total of 50 TBA) knew that caesarean section, and/or exclusive breastfeeding are among the preventive strategies to reduce MTCT of HIV. Here also, no statistical difference was found between trained and untrained TBAs.

In general, TBAs were willing to expand their scope of work regarding mother-and-child activities related to PMTCT. Table 5 shows the activities related to PMTCT that TBAs would agree to participate in. Among interventions related to the pregnant women, more than 75% were willing to participate in all the activities that constitute the basic package of PMTCT services with the exception of blood testing. Less than 25% agreed to accompany the child to the health centre for medication or assist in the documentation of ANC services with the health centres. In this sense, more than 85% of women, both whom delivered with a TBA and with a skilled health worker agreed that TBAs could participate in all activities related to a PMTCT programme with the exception of performing a blood test for HIV for which only 60% and 53% respectively agreed upon (p = 0.10). With regard to pregnant women, older age, lower education level, being of an apostolic faith and having a lower level of knowledge on basic PMTCT concepts were significantly associated with intending to deliver with a TBA (p = < 000.1).

**Table 5:** PMTCT activities that TBAs would agree to participate in TBA survey, Murewa and Goromonzi districts, Zimbabwe, 2006 (n = 72)

	**Traditional birth attendant (TBAs) †**		
		**Agree**	
		
	**Report performing it**	**< 25%**	**> 75%**
**Mothers**			
-Raise awareness			*
-Inform women/men of the benefits of HIV testing			*
- Refer women for HIV testing	**14%**		*
- *Perform blood test*		*	
- Dispense the medication to the women			*
- Directly observe the women ingest the medication during labour			*
- Inform women on appropriate breastfeeding measures			*
- Provide psychological support to HIV + women			*
- Provide continuum of care to HIV + mother and child			*
**Child**			
- Refer the infant to a health centre for medication			*
- *Accompany infant to the health centre for medication*		*	
- Provide medication to the infant			*
			
***Assist health centre in the documentation of ANC services***	**16%**	*	

† All comparisons between trained and untrained TBAs are not statistically significant

ANC: antenatal care

### Focus group discussions

Four focus group discussions (FGDs) were conducted each with a different group of participants: 1) 11 untrained TBAs; 2) eight trained TBAs; 3) eight women who had delivered at a health facility and; 4) eight women who had delivered at home. FGDs with untrained TBAs showed that religious beliefs were part of the guidance of their daily practices. Women of the same religion approach them for services. They highlighted that women who deliver with them are women who have not had complications in previous pregnancies and also women who have financial and/or transport constraints to get to the health centre. All participants confirmed that the high cost of living is forcing most women to deliver outside health institutions as expressed in the following statement:

"Things are hard these days. Where can one get the millions of [Zimbabwean] dollars for clinic fees?"

Traditional birth attendant highlighting the reason why women come to them for delivery

Trained TBAs confirmed that they performed as assistants of the health centre personnel in emergency cases only, when the woman is unable to get to the hospital. In most cases, they do not provide any assistance to women before delivery but encourage them to book their pregnancies for antenatal care. A TBA explained:

"If she comes to me whilst there is still time, I will accompany her to the health centre." "I accompany her so that I can assist along the way in case she may deliver before we get to the health centre."

Traditional birth attendant talking about her tasks with pregnant women

TBAs did not deliver women who had previous pregnancy complications and also those with first pregnancies. TBAs felt that women should better prepare themselves for any problems by booking their pregnancies early with the health centre. Concerning knowledge on basic HIV/AIDS issues, TBAs interviewed knew the existence of HIV, however not all had heard about PMTCT. They recognized that they never suspected any woman of being HIV-positive but only knew about it later when the woman fell sick and died. They said they would not change their attitude towards delivering women who were HIV-positive but that they will need support from the health centres with supplies such as gloves so as to protect themselves.

These community workers confirmed their willingness to participate in PMTCT interventions but recognised the need for training, to agree on what services would be delivered and the necessity to be somehow recognised by health personnel to avoid problems when providing PMTCT services.

Discussion with women who had delivered at a health facility showed they had done so due to the fact that they had chosen to undertake antenatal visits at a health facility as here, medication is provided. These women return to health facilities for child immunization and are aware of the PMTCT services available at the health centres nevertheless, fear for HIV-testing due to discrimination and stigmatisation was highlighted. As to the participation of TBAs in PMTCT, these women think that not all TBAs would be able to carry out the services, even if they were trained, due to an important number being old.

Concerning women who had delivered with a TBA, qualitative data shows that they had done so mainly due to a lack of means of transport to the health centres when the delivery was an emergency. These women had undergone ANC at the health centres but considered the relationship between the health personnel and themselves to not be satisfactory which discouraged mothers to attend maternal services. One woman said:

"Health personnel do not listen to us when we go to the health centre, whereby TBAs do"

Women who delivered at home with a TBA

As to the TBAs' participation in PMTCT activities, these women think that TBAs are capable of carrying out these services on the condition that they are trained, but they do not think that TBAs are capable of performing the blood test for HIV. In addition, a worry that arose was concerning the ability of the TBA to keep women's HIV-sero-status confidential. One woman who delivered with a TBA commented:

""Now it depends on how the TBAs can keep a secret if I reveal my HIV status. I do not want a situation where every one in the community knows that I am positive. "You know, with the health centre, the nurse does not know you so she does not have anyone to tell,"

Women who delivered at home with a TBA

## Discussion

Current PMTCT programmes do not reach many of the women who need them due to socio-cultural, economic, systemic and programmatic factors [38-40]. Home deliveries by traditional birth attendants are common in rural Zimbabwe and are increasing [19]. Furthermore; PMTCT programmes require complementary approaches to prevent missed opportunities in this evolving context. This report is among the first attempts to evaluate the feasibility and acceptability of the participation of TBAs in PMTCT programmes [33].

TBAs in this rural context are elderly, married or widowed and have a minimum level of education. These socio-demographic characteristics are similar to those of TBAs in other settings [41]. Untrained TBAs were younger, had less experience than trained TBAs and learned to attend births by themselves or by helping another TBA. The decrease in training programmes for TBAs in Zimbabwe could explain for the younger age amongst untrained TBAs.

The integration between community health workers such as TBAs and the formal health services can bring valuable benefits to community-based public health interventions and open ways for a number of activities related to prevention and care as has been shown in other reports [42,43]. Complementary approaches, in which community-based interventions are paired with the strengthening and/or expansion of services at the health facility level, also have the potential to address a variety of other health challenges, such as uptake of HIV testing and compliance to PMTCT regimens [33,44].

Recent studies have alluded to problems associated with home deliveries with reference to uptake of PMTCT interventions [45,46]. The present study has demonstrated that beyond TBAs' current activities which include assistance during delivery and in the postpartum stage, they are willing to expand their scope of work in mother-and-child activities to include PMTCT with some limitations identified such as accompanying the child to the health centre for medication and assisting the health centre in the documentation of ANC services. This data suggests the need to strengthen the health care network system between the formal health services and the communities including TBAs. For this, integrating the services of TBAs into the mainstream health care delivery system is required. The existing health care system will have to build-up a partnership with TBAs who operate in the informal sector and help develop communication skills in the referral process. Furthermore, health authorities and health staff need to recognise the cultural and practical contribution of TBAs to the health system.

The success of community-based interventions that emphasize the participation of TBAs depends on the role and status of TBAs in a given community. In India, a 62% reduction in neonatal mortality was achieved through a community-based approach that included training of TBAs and local women to treat sick newborns at home [47]. Furthermore, a trial in Pakistan reported substantial benefits in the reduction of perinatal mortality and maternal mortality by training and integrating TBAs in the health system [48].

Our results reveal that TBAs in this setting have limited knowledge on HIV/AIDS issues in general and PMTCT in particular. This can be improved through the training of these women who are already available and working in the community. This training will need to be tailored to the tasks that they are expected to perform, the knowledge and skills required and adapting the training curricula to the corresponding education level. A recent meta-analysis of 60 studies indicated that training TBAs was associated with significant improvements in their level of knowledge and the quality of advice they provided on family and child health [49].

The present study adds weight to the need of reinforcing TBA's knowledge on MTCT prevention measures before they can contribute to the provision of PMTCT services. At the moment, in this rural setting, TBAs' advice to women on HIV/AIDS issues (including PMTCT) is not frequent. At national level, there is a need to up-date the national TBA training manual by including basic concepts of HIV prevention [50]. In Tanzania, it has been shown that TBAs, if given additional skills and motivated, can be used effectively in program implementation and contribute to reaching women who deliver outside health facilities with PMTCT interventions (from counselling to providing single-dose nevirapine [sdNVP]) [51]. After careful selection, training and sustained and regular supervision, TBAs played an important role in supporting and referring pregnant mothers for facility-based PMTCT services in Uganda [52].

Performing a blood test was one of the activities TBAs refused to practice in the study. The application of this intervention by community workers such as TBAs is directly related to the national policy of each country and if ever promoted will need to include continuous supervision and follow-up. The first reported PMTCT programme to use TBAs to provide confidential HIV counselling and testing utilizing an oral fluid rapid test has been in Cameroon [32]. TBAs also dispense NVP to HIV positive women and ensure that the newborn receives postpartum NVP prophylaxis as an integrated strategy in the PMTCT programme. This approach has been done through community participation, training and complemented with supervisory nurses who visit villages at monthly basis.

Involving TBAs in PMTCT interventions is supported by various individual reports that have shown improvement of TBAs' effectiveness in the provision of public health interventions [53,54]. A recent systematic review showed that TBA training appears to increase antenatal care attendance rates by 38% [55].

In these two rural districts of Zimbabwe, the success of MTCT prevention programmes could be improved by strengthening community-based programmes, and include TBAs who could: establish links between the community and health services and, provide health education to encourage increased use of ANC services, hence accessibility to PMTCT; sensitize communities aiming at a family-centred PMTCT approach [24,56] including information and communication of basic concepts on PMTCT and the importance of HIV testing for the pregnant women and their partners; provide community-based counselling and testing services for HIV [33,57]; support of and adherence to follow-up of PMTCT regimens [45,50]; provide mother-infant adequate referral [58,59]; counsel women to bring newborns to a health centre for the infant dose of NVP; supervision of home administration of infant dose NVP through single-dose blister packs for newborns [60,61]; support women to adopt safer infant feeding practices and promote and support of family planning and; provide care and support to affected families. The dispensing of medications to women in PMTCT programmes as has been reported elsewhere will strongly depend on the national policy of each country and the availability of an efficient training and supervision system [32,51].

Even though doubts have been stated on the possible significance of health improvements that would be gained by attributing HIV prevention and care tasks to TBAs during home deliveries in developing countries [62,63], our data allow the assumption that using these primary health workers for community health interventions including PMTCT may have a potential improvement of perinatal and maternal health indicators.

Another important finding was a positive association between women who delivered at home and the opportunity they had to choose the place of delivery. Decision-making power, gender inequalities and social pressure especially from spouses and other relatives has been reported to significantly influence the use of maternal and child health care [64,65]. It has been well documented in Africa that women lack the power to make independent decisions with regard to their own health care and that of their children [66]. Even though this study did not explore in detail gender and decision making health issues, we can suggest that measures aimed at encouraging women to deliver in health centres will have to involve men if they are to be successful. Furthermore, socio-economic factors can also explain this situation as educated women tended to be better economically empowered than non-educated women and could take decisions on their own [67].

In this study, many of the women who had delivered with a TBA and TBAs themselves cited cost fees as a major determinant of choice of place of delivery, which is consistent with other findings in similar settings [5,68]. This factor, as well as the quality of care expressed in this study as negative experiences women encounter during their interactions with health workers in previous pregnancies, have been acknowledged as important reasons for the non-use of maternal services including PMTCT services and delivering outside the formal health service [69]. In situations such as the one experienced here where women combine TBAs and professional care and where TBAs encourage women to use ANC service, strengthening existing basic antenatal service delivery in general and before introduction of additional interventions such as PMTCT programmes in particular is needed [39].

Women's cited fear of HIV testing and knowing one's HIV status was due to discrimination and stigmatization or breach of confidentiality. Fear of knowing one's HIV status has been described previously as an important reason for drop out from PMTCT services and low levels of HIV status disclosure [70]. Songok *et al *raised concern that non-compliance to the intrapartum dose of sdNVP in a PMTCT programme in Kenya, was due to mothers giving birth at home and fear of TBAs knowing [71]. Presence of TBAs discouraged them from taking the dose for fear of exposing their HIV status. It is thus important to extend and reinforce partnerships among different stakeholders at health centre and community level to support education and access to health information for all women in particular and the community in general in order to prevent stigma and discrimination. In addition, training, deployment and supervision of community health workers including TBAs, must emphasize the importance of confidentiality and the need to support women in the process of disclosure of their HIV-serostatus. HIV status disclosure may lead to improved access to HIV prevention and treatment programmes, increased opportunities for risk reduction and awareness of HIV risk to untested partners, which can result in greater uptake of voluntary HIV testing and counselling, [72] and adherence to the advise given to prevent postnatal and sexual HIV transmission [73].

Qualitative results shown in the FGDs confirm the willingness of TBAs to participate in PMTCT interventions. Nevertheless, as we found in the community survey, women who had delivered in a health centre or with a TBA agreed that for TBAs to be involved in PMTCT activities they need to be trained.

Several potential limitations should be addressed in conjunction with our findings. First, the sample was limited to two rural districts of Zimbabwe and as so, may not be representative of the whole of Zimbabwe. Nevertheless, the socioeconomic characteristics of women of reproductive age considered in this study reflect those of Zimbabwe and many other African settings. Second, respondents' accounts of subjective events around pregnancy and childbirth may have been prone to recall bias. However, studies have shown that the recollection of various factors related to pregnancy and delivery is accurate, even over long periods [74]. A third consideration relates to possible inconsistencies especially where the respondent could not understand the agreed Shona terminologies as this might have affected the understanding of some questions by the respondents. This was controlled to some extent through training of data collectors on the terminologies to be used for the Shona translations to minimize errors and through piloting of the tools.

## Conclusion

We conclude that when health professionals are not available, TBAs are a significant workforce in maternity care in high HIV burden countries of sub-Saharan Africa such as Zimbabwe. Our findings suggest that in Zimbabwe, although the long-term provision of ANC services should remain in the hands of skilled attendants, encouraging the participation of TBAs in public health programmes could help to increase the coverage of mother-child health care including PMTCT. This approach has a potential to prevent missed opportunities in a context where an important number of pregnant women resort to home deliveries. Furthermore, implementation of more complex regimens for PMTCT as recommended by the WHO and planned by the Zimbabwean PMTCT programme requires not only on site availability of appropriate ARV drugs and the ability to assess the state of HIV disease of identified HIV-infected pregnant women as well as their eligibility for HAART but also monitoring and support for the administration of these short-course regimens [75]. Community health workers can have an important role in the decentralized programmatic implications of adding more complex PMTCT regimens to current basic PMTCT services, mainly with regards to support and drug adherence follow-up. A challenge for policy makers in developing countries is to make the best use of available human resources including TBAs but also to plan and implement a definite public health strategy based on essential obstetric care provided by skilled attendants.

Identifying roles for community workers such as TBAs can facilitate the improvement of mother-child health when strategies such as upgrading existing lower-level facilities, improving and building referral systems, training and supervision are considered. Here, it will be necessary to adopt health policies that do not coerce but rather legitimize and acknowledge the practice of TBAs and integrate them into specific health programmes. There is very limited evidence-based data on community interventions and the impact of community health workers in improving maternal health, including their role in HIV/AIDS interventions. Operational research in this field is still needed since it is likely that in the foreseeable future, community health workers including TBAs could contribute to the uptake and adequate coverage of PMTCT services that are needed to fulfil the MDGs in HIV/AIDS in many countries in sub-Saharan Africa and elsewhere.

## Competing interests

The authors declare that they have no competing interests.

## Authors' contributions

FP: conceived the study, participated in the design, supervised study implementation, undertook analysis and interpretation of the data and drafted the manuscript. DAK coordinated overall survey implementation, data collection and revised the paper critically for substantial intellectual content. TN coordinated district-level survey implementation and revised the paper critically for substantial intellectual content. BE provided technical expertise and revised the paper critically for substantial intellectual content. FD provided inputs to the study design and edited the manuscript. All authors read and approved the final manuscript.

## Pre-publication history

The pre-publication history for this paper can be accessed here:


